# The oldest semi-aquatic beaver in the world and a new hypothesis for the evolution of locomotion in Castoridae

**DOI:** 10.1098/rsos.220926

**Published:** 2022-08-24

**Authors:** Jonathan J. M. Calede

**Affiliations:** Department of Evolution, Ecology, and Organismal Biology, The Ohio State University at Marion, 1459 Mount Vernon Avenue, Marion, OH 43302, USA

**Keywords:** amphibious, Arikareean, astragalus, body size, exaptation, phylogenetic comparative methods

## Abstract

The North American rodent fossil record includes hundreds of species representing both an incredible taxonomic diversity and great ecological disparity. Although it is during the Oligocene that taxonomic diversity first peaks, it is not until the Miocene, almost 10 Myr later, that many ecologies, particularly locomotory ecologies, are recorded. Here, I present a new Oligocene-aged species of beaver from Montana, *Microtheriomys articulaquaticus* sp. nov., which represents the oldest semi-aquatic rodent in North America and the oldest amphibious beaver in the world, pushing the advent of semi-aquatic ecology in beavers by 7 Myr. I also provide morphological data supporting a terrestrial ecology for the sister taxon to Castoridae. Together with existing data, these findings lead to a new hypothesis for the evolutionary ecology of castorids whereby swimming was exapted from burrowing during the Oligocene. This evolution of semi-aquatic locomotion may have taken place in North America instead of Eurasia. It started in small beavers with gigantism achieved only much later. Indeed, body size evolution in castoroids follows a directional drift. Beavers obey Cope's rule, a selection for larger size over time that appears associated with semi-aquatic ecology and may well explain their low modern diversity.

## 1. Introduction

The incredible taxonomic diversity of Rodentia, the most species-rich order of mammals, is associated with an amazing ecological disparity [1–4]. One aspect of this ecological range is found in locomotion; there are gliding, burrowing, cursorial, arboreal, terrestrial and semi-aquatic rodents [3,5]. Semi-aquatic ecology has evolved several times within Rodentia; semi-aquatic species exist within the families Cricetidae, Muridae, Echimyidae and Castoridae [5,6]. In North America, four extant native rodents are semi-aquatic. Three belong to the family Cricetidae, *Ondatra zibethicus* (the muskrat), *Neofiber alleni* (the round-tailed muskrat) and *Oryzomys palustris* (the marsh rice rat); one to the family Castoridae, *Castor canadensis* (the American beaver). All four of these taxa are first recorded in the fossil record during the Pleistocene [7]. The Cenozoic fossil record shows evidence for the presence of over 35 species of semi-aquatic rodents during the Miocene and Pliocene, particularly within the family Castoridae (=beavers) [7]. The oldest rodent species in North America whose postcranial morphology supports a semi-aquatic ecology, the beaver *Monosaulax baileyi*, is 18.8 Myr old [7,8]. The oldest semi-aquatic beaver in the world, *Steneofiber eseri*, dates back to the Miocene; it is 23 Myr old [9]. Based on taxonomic affinities, older beavers have been suggested to have been semi-aquatic [10,11], but no morphological evidence unequivocally supports these hypotheses. The timing and pattern of evolution of the semi-aquatic ecology found in extant beavers is particularly important to decipher because the fossil record of Castoridae shows that, for much of their early evolution in North America, many species were in fact burrowing [5,8,10]. Indeed, the subfamilies Palaeocastorinae and Migmacastorinae, which evolved in North America, include almost 20 species, some of which would dig very complex burrows [12]. Burrowing beavers are absent outside of North America [10], but there is no evidence for semi-aquatic beavers until the Miocene and the locomotion of many early beavers has either been considered equivocal or representative of a terrestrial ancestry [10]. The sister family to Castoridae, the Eutypomyidae also appear to have been terrestrial [10], although no rigorous analysis of their locomotion has ever been undertaken. Here, I present the first postcranial remains for the Oligocene-aged beaver *Microtheriomys*. These include the astragalus, an ankle bone that enables the rigorous test of the hypothesis that semi-aquatic castorids evolved during the Oligocene.

The morphology of ankle bones, including the astragalus, has made possible the study of mammalian locomotion [13]. The shape of ankle bones is associated with locomotion and habitat use in ungulates [14,15], carnivores [16–18] and primates [19,20]. Numerous studies have also been undertaken in rodents, providing insights into the locomotory ecology of numerous clades through space and time [21–27]. I expand upon this work and present a database of astragalar measurements for over 135 rodent species that enables the exploration of morphological proxies of locomotion using phylogenetic comparative methods. I use these data to infer locomotion in *Microtheriomys*. I also formally constrain the locomotion of the sister taxon to beavers, Eutypomyidae. These data provide the necessary information to explore the evolution of locomotion in Castoroidea. I test the hypothesis that an ancestrally terrestrial ecology gave rise to both burrowing (Palaeocastorinae) and semi-aquatic (including Castorinae, Castoroidinae and Anchitheriomyinae) beavers with similar morphological adaptations.

Existing data on castoroid rodents make possible an evaluation of the evolution of beaver body size through time and its possible associations with (i) the evolution of locomotion and (ii) changes in biodiversity within Castoridae. Indeed, aquatic locomotion has been demonstrated to lead to increased body size in several mammalian lineages [28]. It has also been suggested that increases in body size following Cope's rule can lead to decreased biodiversity [29]. I specifically test the hypothesis that castoroid body size follows a positive shift, which would support Cope's rule, associate patterns of increasing body size with the evolution of an amphibious ecology, and possibly explain the collapse of beaver diversity.

## 2. Material and methods

### 2.1. Stratigraphic context of *Microtheriomys* specimens

The fossil material described includes a specimen composed of several associated teeth, two astragali and partial bones (UMPC 947), which I describe as the holotype of a new species. Along with other isolated teeth, partial jaws and a crushed juvenile cranium, it was recovered from fluviolacustrine deposits of the lower unit of the Arikareean-aged Cabbage Patch beds (Renova Formation) in Montana. Another specimen (UWBM 108110) is from the middle unit of the beds. The age range for the new taxon is 29.92 ± 0.07 Ma to 27.05 ± 0.26 Ma [30].

### 2.2. Sampling and phylogeny

I included 343 rodent specimens in the training set of my analyses of rodent ecomorphology (table 1). Of these, 313 specimens represent 127 extant species from 104 genera and 28 families (=80% of rodent families). They include a wide range of body sizes (electronic supplementary material, S1), overlapping the size of *Microtheriomys* in all eight locomotory categories (table 1). The locomotion for these taxa was taken from prior analyses of rodent locomotion and natural history accounts, including data from congeneric species (electronic supplementary material, S1). I follow prior studies of rodent ecomorphology for the definitions of locomotor groups [5,8] in restricting semi-aquatic rodents to hind limb and tail paddlers (excluding for example the capybara, *Hydrochoeris hydrochaeris*, categorized instead as cursorial) [8,31]. Unlike a previous analysis of astragalar shape in rodents [21], I used a narrow ‘jumping rodent’ category restricted to ricochetal (i.e. bipedal saltatorial) animals, differentiated between semi-fossorial and fossorial taxa, and isolated gliding taxa from arboreal ones.

**Table 1.** Distribution of the training set across locomotory categories.

locomotion	extant rodents		fossil beavers	
	species	specimens	species	specimens
arboreal	21	49	—	—
cursorial	6	18	—	—
fossorial	21	49	3	5
gliding	13	26	—	—
ricochetal	8	19	—	—
semi-aquatic	10	27	9	25
semi-fossorial	18	46	—	—
terrestrial	30	79	—	—

Extant castorids are known from only two species from a single genus (both included in the training set) and represent a small subset of the morphological disparity observed in the family [8,12]. To better represent the ecomorphologies and body sizes of beavers, I included 30 specimens of fossil castorids representing 12 species and eight genera in the training set (table 1). The *a priori* locomotion of these fossil taxa was taken from previous analyses (electronic supplementary material, S1).

In addition to the specimen of *Microtheriomys* from the Cabbage Patch beds of Montana (UMPC 947), I also analysed the paratype of *Eutypomys thomsoni* (AMNH 12255) [32,33]. *Eutypomys* is a member of the extinct family Eutypomyidae, the sister taxon to the family Castoridae [10,34] which is assumed to be terrestrial [10], but empirical support for this locomotion is lacking.

The phylogenetic framework used in the phylogenetically informed flexible discriminant function analysis (pFDA) (see below) was assembled from a time-calibrated tree of the extant species [35] and a time-calibrated supertree of beavers. For the extant dataset, I randomly selected 100 trees from a published selection of 1000 time-calibrated trees [36], pruned the trees to keep only the 313 tips with morphological data and randomly resolved all polytomies using the R package ape 5.3 [37]. For the fossils, I used a published phylogeny [10,38] and taxonomic data [34] to build a supertree of the beavers included in the training set (figure 2*a*). *Microtheriomys* was added to this tree based on its previously published taxonomic affinities [11]. I time-calibrated the supertree of fossil castorids using the R package strap 1.4 [39], first appearance data (FAD) and last appearance data from the supplementary information file of Samuels & Hopkins [7], and the ‘equal’ approach. The root length of 1.9 was chosen based on the difference between the FAD of *Eutypomys thomsoni*, the oldest ingroup taxon in the analysis (33.9 Ma) and the FAD (35.8 Ma) of the next oldest taxon within the outgroup to Castoroidea (Geomorpha + Eomyidae) [40]: *Montanus bjorki* [7]. The castorid supertree was then inserted in the phylogeny of extant rodents using ape. The tree used in the analyses is available in the electronic supplementary material.

### 2.3. Ankle morphology and locomotion

Morphological data for 43 specimens were taken directly from a prior study [21]. Five specimens were measured from published illustrations [27,40]. Data for the remaining 297 specimens were measured following published analyses [21]. Measurements were only collected from fully fused specimens using specimen photos and ImageJ 1.51 [41] or directly under a DinoLite AM7915MZT digital microscope to ensure the correct orientation of the specimen and the repeatability of the measurements. I did not include in the analyses the length of the neck of the astragalus because the measurement is difficult to replicate, retaining 15 measurements per specimen. All measurements were divided by the geometric mean (GM) for the specimen [42,43] and log transformed. Mean measurements were calculated for each species. Training set data were input into a jackknifed canonical variate analysis using the R package MASS 7.3-51.5. The two target fossils were added *a posteriori* to determine their likely locomotion.

I also used a pFDA to investigate the relationship between astragalar morphology and locomotion [3,44–46]. This is because the long evolutionary history of rodents could have played an important role in shaping their ankle and the sample's distribution across the rodent tree is not random. I modified published scripts [3,44] and used the packages ape and mda in R to calculate Pagel's lambda for all 100 supertrees. These lambda values were input into the pFDA, iterated for all 100 trees. The posterior probabilities for the locomotion predictions for all 100 analyses for both *Microtheriomys* and *Eutypomys* enable locomotion inferences*.* I used the results of the pFDA and maximum-likelihood implemented in the R package ape 5.3 [37] to reconstruct ancestral character states for locomotion at the nodes for the entire sample for a single random tree. A single-tree analysis was sufficient because of the unique topology of the castorid phylogeny. There are no significant differences in the locomotory evolution scenarios within Castoridae across the 100 trees.

### 2.4. Body size

The body mass of fossil mammals can be assessed using a number of osteological and dental proxies [47–49], including measurements of the astragalus [50] and toothrow length [51]. I used astragalus GM to compare the body size of *Microtheriomys* with 19 modern and fossil semi-aquatic taxa included in the locomotion analysis. Because fossil beaver astragali are rare, I also used published lower toothrow lengths (LTRL) to compare the body size of *Microtheriomys* with 75 extinct castorids with known locomotion and the two extant beavers [5,8,12,52]. When necessary, I used the relationship between upper and lower toothrow lengths to estimate LTRL [52]. LTRL was measured from three complete lower dentitions of adult specimens of *Microtheriomys* (UMPC 2482, KUVP 18171 and UWBM 108110) and calculated for the holotype (UMPC 947) by adding the lengths of the associated cheek teeth.

The mean LTRL of *Eutypomys thomsoni* was calculated from published values [33]. All proxies were log transformed prior to analyses. I used the published regression for non-muroid rodents under 5 kg to estimate the body mass of *Microtheriomys* [51]. The LTRL of all 16 castoroid taxa for which locomotion is known (or inferred herein) is used to reconstruct the evolution of body size in the clade using an ancestral character state reconstruction [53,54] and model fitting in the R package geiger 2.0.7.1 [55]. I tested five models of trait evolution (Brownian motion, early-burst, rate trend, directional drift and Ornstein–Uhlenbeck) and determined the best fit using Akaike weights. The analyses were run for a single random tree for the same reasons as the ancestral character state reconstruction of locomotion.

## 3. Systematic palaeontology

Class Mammalia Linnaeus, 1758 [56]

Order Rodentia Bowdich, 1821 [57]

Family Castoridae Hemprich, 1820 [58]

Subfamily Anchitheriomyinae Korth, 2001 [59]

Genus *Microtheriomys* Korth and Samuels 2015 [11]

Type species *Microtheriomys brevirhinus* Korth and Samuels, 2015, Arikareean 1; John Day Formation, Turtle Cove Member, Oregon.

### 3.1. New species

**Figure 1.**
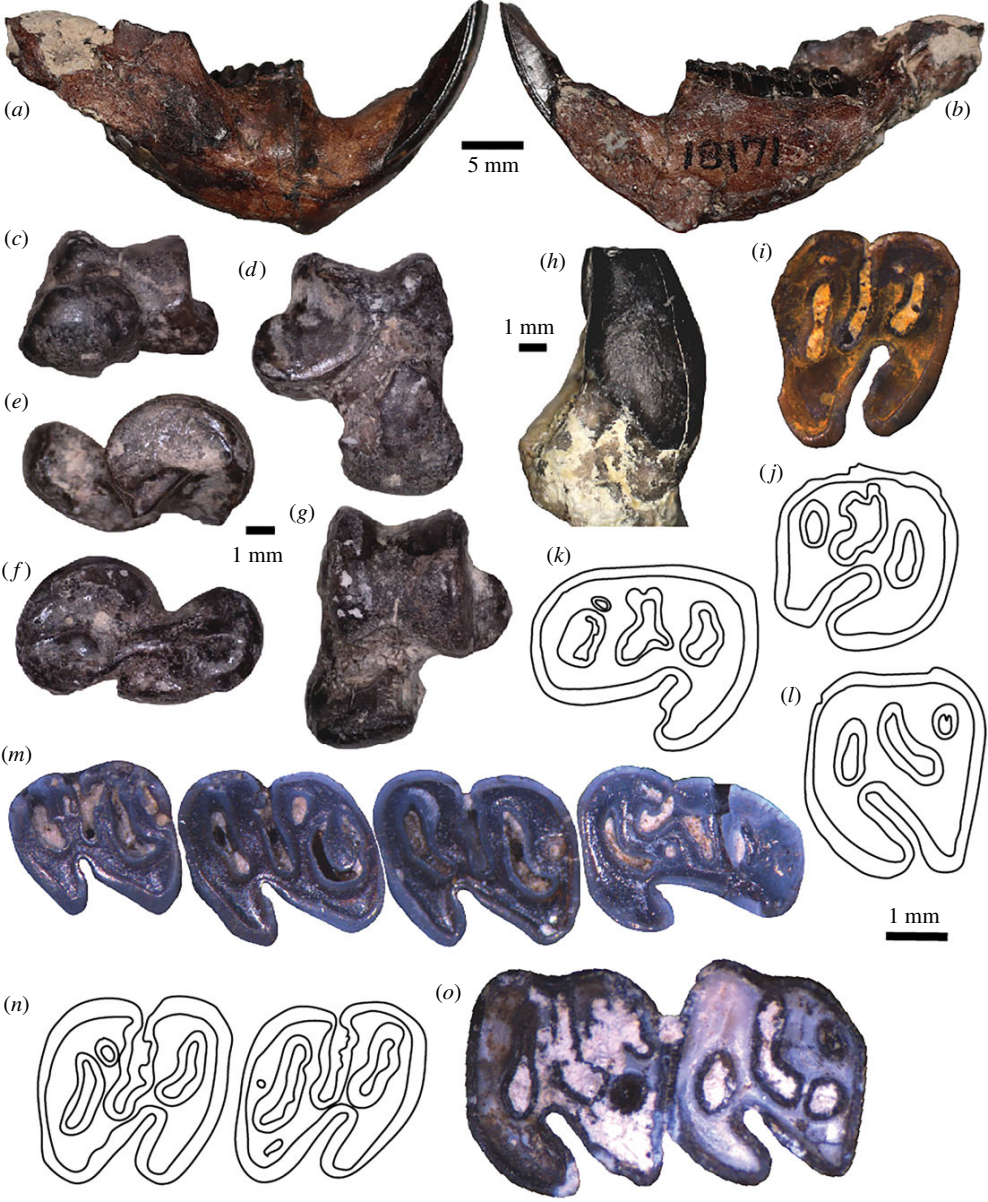
Morphology of *Microtheriomys articulaquaticus* sp. nov*.* KUVP 18171 (paratype), dentary: (*a*) lateral view, (*b*) medial view; UMPC 947 (holotype), left astragalus: (*c*) anterior view, (*d*) plantar view, (*e*) lateral view, (*f*) medial view, (*g*) dorsal view; UWBM 97500, M1: (*h*) anterior view; (*i*) UWBM 101347, M1/2; UMPC 947 (holotype), select teeth: (*j*) RM2, (*k*) Lp4, (*l*) LM1; KUVP 18171 (paratype): (*m*) p4-m3; (*n*) UMPC 2260, m1-2; (*o*) UWBM 98741, M1-2. Bottom right scale is for all occlusal views.

*Microtheriomys articulaquaticus* sp. nov. (see figure 1).

### 3.2. Synonymy

*Microtheriomys* new species A (Calede 2020).

### 3.3. Etymology

Named for the Latin for joint (*articulus*) and for aquatic (*aquaticus*) to reflect the locomotory ecomorphology determined from the ankle bone of this animal.

### 3.4. Material

Holotype: UMPC 947 (figure 1), partial skeleton with isolated partial LP4, LM1 or M2, partial LM3, RM2, Lp4, Lm1 or m2, Lm3, R m1 or m2. Paratype: KUVP 18171 (figure 1), partial right dentary with I, dp4, m1-m3. Referred specimens: UMPC 2482, partial right dentary with i, p4-m2; KUVP 18842, partial left dentary with dp4, p4, and m2-3; UMPC 1484, left m1; UMPC 1487, left dp4; UMPC 1493, right m3; UMPC 1535, right P4; UMPC 1544, right p4; UMPC 2199, partial skull with left DP4-M2, partial right P4-M2, and partial left dentary with I, dp4-m3; UMPC 2213, right m1 or m2; UMPC 2259, partial left dentary with m1-2; UMPC 2260, partial left dentary with m1-2; UMPC 3028, right m1-3; UMPC 13212, m3; UWBM 97359, M3; UWBM 97500, partial maxilla with M1-2; UWBM 98741, partial maxilla with M1-2; UWBM 101347, M1 or M2; UWBM 108110, dentary with p4-m3, associated crushed skull and M1-2.

### 3.5. Locality and horizon

Lower and middle units of the Cabbage Patch beds, Renova Formation, Montana, USA; early Arikareean, Oligocene.

### 3.6. Diagnosis of species

Much smaller than *Anchitheriomys*, *Oligotheriomys* and *Miotheriomys*; only slightly smaller than *M. brevirhinus*; rostrum shorter than other anchitheriomyine genera; incisors with rugose enamel surface, but not ridged like *M. brevirhinus*, but unlike *Anchitheriomys* which bears ridges; P3 absent unlike in *Agnotocastor* and *Oligotheriomys*; cheek teeth unilaterally hypsodont and upper toothrows parallel to one another like in *M. brevirhinus*, but unlike *Propalaeocastor*; P4 much larger than M1-2 unlike in *M. brevirhinus*; cheekteeth lower crowned than *M. brevirhinus*; upper molars generally wider than long unlike *M. brevirhinus*; occlusal pattern of cheekteeth less complex than in *M. brevirhinus*.

### 3.7. Brief description of the fossils

*Microtheriomys articulaquaticus* sp. nov. is a smaller beaver than *M. brevirhinus*. A crushed juvenile skull shows several characteristics of the genus including a short rostrum, parallel upper toothrows, and the absence of a P3. Additionally, the cheekteeth are unilaterally hypsodont and display a complex pattern of enamel lakes (=fossettes and fossettids; see electronic supplementary material for tooth nomenclature) (figure 1). The P4 is much larger than M1, to a greater extent than in *Microtheriomys brevirhinus* [11]. The shape of the upper cheekteeth varies across and even within specimens although they are generally wider than long, unlike in *M. brevirhinus* (electronic supplementary material, table S1). The DP4 greatly resembles the P4. The only difference between the two teeth, other than size and proportion, is in the connection of the parafossette with the mesoflexus in the DP4, which is absent in P4. The occlusal surface of the P4 of *M. articulaquaticus* sp. nov. differs from that of *M. brevirhinus* in the shape of the parafossette, which is more buccolingually elongated in *M. brevirhinus*, and a more complex distobuccal corner of the tooth in *M. articulaquaticus* sp. nov*.* The two species are similar in the morphologies of the anteroflexus, anterofossette and mesoflexus. The M1 is much wider than it is long (electronic supplementary material, table S1). There is little mesiodistal compression of the four main cusps (paracone, protocone, metacone and hypocone). The morphology of M2 is very similar to that of M1. In general, the morphologies of M1-2 in *M. articulaquaticus* sp. nov. is simpler than that of *M. brevirhinus*. The shape of the enamel lakes on the occlusal surface of M3 is very complex. The tooth bears a very large protocone.

The dentary is dorsoventrally shallow in both juvenile and adult specimens (figure 1). The root of the incisor terminates posteriorly to m3 and the diastema is short. The mandibular symphysis is strong and extends from the edge of the incisor alveolus posterior to a point ventral to the p4-m1 contact, including the digastric process. The mental foramen is single, round and small in the paratype, where it is located ventral to the anterior edge of p4 (figure 1). The masseteric fossa is dorsoventrally deep and ends ventral to m1. The coronoid process ascends posteriorly to m1. The lower incisor is relatively narrower in *Microtheriomys articulaquaticus* sp. nov. than in *M. brevirhinus* (electronic supplementary material, table S2) and bears a faint ornamentation on the anterior surface. The dp4 is more mesiodistally elongated than in *Microtheriomys brevirhinus* (electronic supplementary material, figure S1); so is the p4 (electronic supplementary material, table S2). The lower molars tend to be squarer than in *M. brevirhinus*. The width is sometimes greater than the length, unlike in *M. brevirhinus*. The morphologies of m1 and m2 are very similar. The m3 differs from the anterior molars by a more buccolingually compressed talonid and broader metafossettid (electronic supplementary material, figure S2).

The caudal vertebrae are short and round in cross-section; just like in many other semi-aquatic fossil castorids [60], there is no evidence of flattening, unlike in *Castor* (electronic supplementary material, figure S3). The appendicular skeleton is represented by a partial proximal left radius, a partial distal right tibia, a complete left astragalus (figure 1), the body and fragmented neck and head of the right astragalus, a partial left calcaneus, a right navicular, partial left navicular, a right cuboid, an entocuneiform, and several metapodials and phalanges (electronic supplementary material, figure S3). The proximal left radius resembles greatly a miniaturized version of the radius of *Castor* [61]. The distal tibia also bears a strong resemblance to that of *Castor*. The depression for the head of the astragalus in the navicular is shallower than in *Palaeocastor* [38]. The astragalus of *Microtheriomys* resembles the astragali of semi-aquatic rodents like *Castor* and *Procastoroides* [38,60,61] in several characteristics: (i) both the medial and lateral trochlea are dorsoventrally short; the sulcus between them shallow, (ii) the body of the astragalus is wide because of a laterally expended astragalofibular facet and (iii) the lateral trochlea is anteroposteriorly shorter than the medial one. The shape of the head of the astragalus resembles that of *Castor.* The sustentacular and ectal facets are separated by a wide sulcus also as in *Castor*. The ectal facet of the astragalus of *Microtheriomys* is most similar to *Dipoides* and *Eucastor malheurensis* [60]. The sustentacular facet contacts the navicular facet and is mediolaterally wide as in many semi-aquatic castorids*.* The plantar process of the sustentacular facet [21], a characteristic of Castoridae is present, unlike in *Palaeocastor* [38]. Overall, the navicular of *Microtheriomys* resembles most that of *Procastoroides*. The calcaneus is only partially preserved. Overall, its shape is more alike those of *Castor* than *Palaeocastor* or castoroidine beavers [38,60,61]. The sustentacular facet resembles most closely the sustentacular facet of the calcaneus of *Eucastor malheurensis* [60]. The ectal facet differs little in shape from several other semi-aquatic beavers across both Castorinae and Castoroidinae [60]. The entocuneiform of *Microtheriomys* most closely resembles that of *Dipoides* whereas the cuboid is more similar to that of *Castor* or *Eucastor* [60]. The metatarsals resemble most those of *Dipoides* [62] or *Procastoroides* [60]. The distal phalanges resemble most those of *Castor* [60]. A detailed description of *Microtheriomys articulaquaticus* sp. nov. is provided in the electronic supplementary material.

## 4. Results of quantitative analyses

### 4.1. Ankle morphology and locomotion in extant rodents

**Figure 2.**
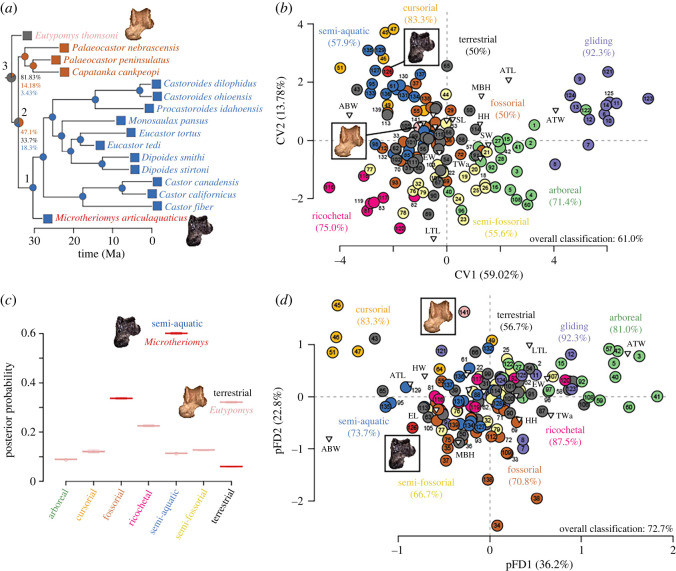
Analysis of locomotory ecology of *Microtheriomys* and *Eutypomys*. (*a*) Supertree of Castoroidea used in the analysis with reconstructed ancestral character states displayed (labelled nodes described in text, likelihoods shown next to pie charts), (*b*) results of the canonical variate analysis of astragalar morphology, (*c*) distributions of posterior probabilities of each locomotion for both fossil taxa obtained from pFDA and (*d*) results of the pFDA. Percentage of correctly classified taxa based on jackknifed analyses shown in both (*c,d*). Colours correspond to locomotion or fossil taxon. Numbers associated with electronic supplementary material, S1. Triangles show eigenvectors. Abbreviations follow Ginot *et al.* [21].

A MANOVA demonstrates that the different locomotory modes differ significantly in morphospace occupation (*F* = 9.4799, *p* < 0.0001). ANOVAs and *post hoc* Tukey tests for individual axes show that semi-aquatic rodents differ significantly in morphospace occupation from all other locomotory categories studied across the first four axes of the CVA. The first two axes of the CVA represent a total of 72.8% of the variance (figure 2*b*). The first axis (CV1) is positively correlated with astragalus total width (ATW), and to a lesser extent astragalus total length (ATL), and negatively correlated with astragalus body width (ABW). CV1 distinguishes gliding and arboreal taxa at the positive end of the axis from species bound to a terrestrial substrate (fossorial, ricochetal, terrestrial, semi-fossorial, cursorial and semi-aquatic rodents) at the negative end. The second axis (CV2) is positively correlated with ATL and medial body height (MBH) and negatively correlated with lateral trochlear length (LTL). CV2 separates cursorial, fossorial, semi-aquatic and terrestrial taxa at the positive end of the axis from ricochetal, semi-fossorial and arboreal taxa at the negative end.

The CVA shows that species can be accurately classified *a posteriori* into locomotory categories 61% of the time (figure 2*b*). Three locomotory modes show particularly good classification rates: gliding, cursorial and ricochetal. Terrestrial, semi-fossorial and fossorial rodents show lower classification rates. The mean posterior classification rate is 7.6 times higher than the prior classification rate (median 4.5).

All one hundred iterations of the pFDA yielded a lambda of 0.1. There are very few differences between the one hundred iterations of the pFDA. The ordinations are similar; one of them is displayed in figure 2*d*. The first two axes of the pFDA represent a mean of 58.9% of the variance (range 58.7–59.1). The first axis (pFD1) is positively correlated with ATW, and to a lesser extent astragalus trochlear width and ectal facet width; it is negatively correlated with ABW. Similarly to CV1, pFD1 distinguishes gliding and arboreal taxa at the positive end of the axis from species bound to a terrestrial substrate at the negative end. Cursorial taxa have the most negative scores along pFD1. The second axis (pFD2) is positively correlated with LTL and negatively correlated with MBH and ABW. Fossorial taxa occupy the negative end of pFD2; cursorial taxa occupy the positive end of the axis. pFD3 (not figured, mean 18.39% of variance) differentiates gliding rodents from other taxa; pFD4 (mean 9.32% of variance) distinguishes ricochetal taxa and pFD5 (mean 6.96% of variance) distinguishes semi-aquatic taxa. pFD5 is positively correlated with LTL and negatively correlated with ABW and EL. Semi-aquatic taxa occupy the negative end of pFD5.

The pFDA accurately classifies 72.7% of the species studied *a posteriori* (figure 2*d*). Six locomotory modes have classification rates above 70%: gliding, ricochetal, cursorial, arboreal, semi-aquatic and fossorial. Terrestrial taxa have the lowest rate of correct *a posteriori* classification (56.7%). All classification rates are equal or higher in the pFDA than in the CVA. The mean posterior classification rate is 8.4 times higher than the prior classification rate (median 5.4).

### 4.2. Locomotory inferences of fossil taxa

*Microtheriomys* is classified by the CVA as semi-aquatic with a posterior probability of 85.6%. It is closest in morphospace to the extant Eurasian beaver, *Castor fiber*. The second highest posterior probability is for a fossorial locomotion with 12.0%. For *Eutypomys*, the highest posterior probability is for a terrestrial locomotion (80.2%), the second highest posterior probability is for a semi-fossoriality (9.2%).

The distribution of posterior probabilities across all one hundred pFDAs (figure 2*c*) supports a semi-aquatic locomotion for *Microtheriomys* (mean posterior probability: 60.1%); the second most probable locomotion inferred is fossoriality (mean posterior probability: 33.7%). A semi-aquatic locomotion is the most probable inference in all one hundred analyses. For *Eutypomys*, the highest posterior probability is for a terrestrial locomotion (mean 32.1%), the second highest posterior probability is for a ricochetal locomotion (22.5%). All one hundred analyses support terrestriality as the most probable locomotory ecology.

The ancestral character state reconstruction supports a semi-aquatic locomotion for the common ancestor of Anchitheriomyinae, Castorinae and Castoroidinae (node 1, figure 2*a*). The ancestor of Castoridae (node 2, figure 2*a*) is reconstructed as most likely fossorial. The ancestor of Castoroidea (node 3, figure 2*a*) is reconstructed as terrestrial.

### 4.3. Body size of *Microtheriomys articulaquaticus*

**Figure 3.**
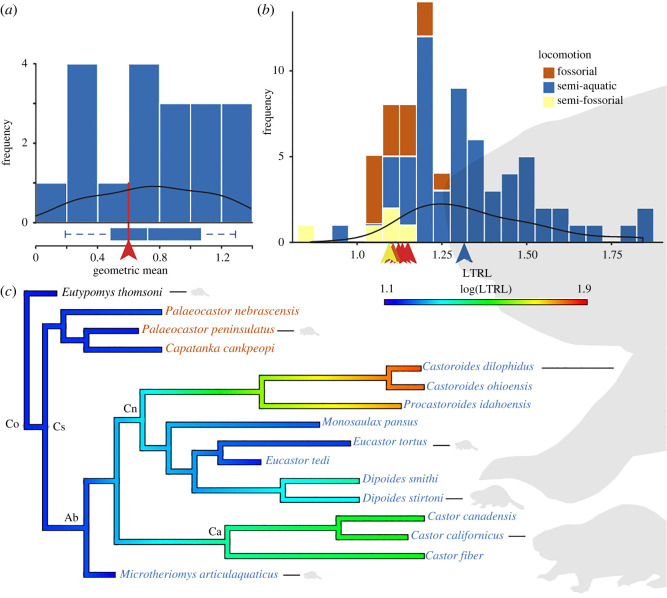
Body size analysis of *Microtheriomys*. (*a*) Frequency distribution of astragalus size (based on GM) across semi-aquatic rodents. The red arrow and line show the size of *Microtheriomys.* (*b*) Frequency distribution of the size (based on LTRL) of extant and extinct castorid taxa. Blue, yellow and brown arrows show the median size for each locomotory category. Red arrows indicate sizes of *Microtheriomys* specimens. Black lines denote density curves for semi-aquatic taxa in both (*a*,*b*). (*c*) Ancestral character state reconstruction of body size as log(LTRL) in Castoroidea. Scaled silhouettes of *Castor* (from Phylopic Public Domain Dedication 1.0 license) linked to select taxa denote relative size (as body mass in grams) for ease of comparison. Note the very large size of *Castoroides*. Abbreviations for clades: AB, amphibious beavers; Ca, Castorinae; Cn, Castoroidinae; Co, Castoroidea; Cs, Castoridae.

The astragalus of UMPC 947 is smaller than the median astragalus of all semi-aquatic rodents included in the locomotion analysis (figure 3*a*); it is also smaller than all other castorids included in the locomotion analysis. The largest semi-aquatic rodents included in the analyses are the extinct castorids *Procastoroides* and *Castoroides*; the smallest are the extant cricetids *Oryzomys*, *Ichthyomys*, *Neofiber* and *Nectomys*. *Microtheriomys* (LogGM: 0.60) is most similar in size to the extant rakali (or Australian water-rat) *Hydromys chrysogaster* (LogGM: 0.57; *N* = 3) and the extant muskrat *Ondatra zibethicus* (LogGM: 0.63; *N* = 3). About 32% of the semi-aquatic rodent species studied are smaller than *Microtheriomys*.

The three specimens of *Microtheriomys articulaquaticus* sp. nov. with complete toothrows or alveoli (KUVP 18171, UWBM 108110 and UMPC 2482) measure 14.17, 14.08 and 13.60 mm, respectively. These measurements correspond to body mass estimates ranging from 696 to 774 g. The holotype (UMPC 947) is estimated at a mass of 597 g based on a reconstructed LTRL of 12.82 mm. The logged median LTRL of *Microtheriomys* (1.14) is smaller than the median LTRL (1.32) of 59 semi-aquatic castorids (figure 3*b*). Only four species of semi-aquatic beavers (out of 64 studied) are smaller than *Microtheriomys*: *Microdipoides eximius*, and three species of the genus *Monosaulax* (*M. progressus, M. typicus* and *M. valentinensis*). *Microtheriomys* is larger than the median LTRL of fossorial castorids and the median LTRL of semi-fossorial castorids (the latter category is only represented by the genera *Capacikala* and *Pseudopalaeocastor*).

The common ancestors of all castoroids, all castorids, all semi-aquatic beavers and the common ancestor of castoroidines and castorines were all small animals (figure 3*c*). The ancestral character state reconstruction of body size provides evidence for the independent evolution of large size in both the Castorinae and the Castoroidinae. Gigantism is achieved independently in *Castoroides* and *Procastoroides*. The best-supported model of body size evolution is overwhelmingly directional drift (Akaike weights—directional drift: 0.975, trend: 0.011, Brownian motion: 0.009, Ornstein–Uhlenbeck: 0.002, early-burst: 0.003).

## 5. Discussion

The new fossil species described herein, its geological context and the comparative analysis of the morphology of its ankle bone (astragalus) demonstrate that the oldest semi-aquatic beaver in the world is *Microtheriomys articulaquaticus* sp. nov., which is first recorded *ca* 30 Ma in North America. Together with new data on Eutypomyidae, the sister taxon to the family Castoridae, this new fossil provides a unique opportunity to explore the evolution of locomotion in Castoridae and its links with body size evolution.

Broadly, the ecomorphological analyses provide evidence that astragalus morphology can be used to classify rodent species in the appropriate locomotory category, particularly in select locomotory modes with distinctive astragalar morphologies: gliding, cursoriality and ricochetal locomotion. Locomotory modes that require little specialization of the hindlimbs (e.g. terrestriality and fossoriality) show lower accurate classification rates. The expanded training set of my analysis shows a much greater accuracy in classifying taxa in their locomotory categories than previous work [21] and can be used to infer the ecology of a wide range of extinct rodent species across body masses, clades and locomotion (electronic supplementary material, S2). My analyses show that semi-aquatic rodents in general are characterized by an astragalus with a wide body, a short trochlea and a long ectal facet. The wide body is a consequence of an expanded astragalofibular facet, which serves as support for the distal end of the fibula, particularly during the eversion of the pes [24]. A relatively stable upper ankle joint facilitates swimming [63]. The short trochlear area of the lateral tibial facet is evidence of a stable cruropedal joint with limited anteroposterior movement [24], once again facilitating hindlimb paddling. In general, semi-aquatic rodents display asymmetrical trochleae and a craniomedially oriented astragalar neck and head, two characteristics consistent with swimming in rodents [25]. In *Microtheriomys* specifically, the deep groove for the calcaneofibular ligament is further evidence of a stable joint [24]. The long sustentacular facet would have enabled a fair degree of rotation between the astragalus and calcaneum; the wide sulcus between the ectal and sustentacular facets would have provided a strong ligament connection between the astragalus and calcaneus, stabilizing this movement of the ankle joint. The extended and strongly concave ectal facet, associated in some taxa like *Microtheriomys* and *Castor* with a long sustentacular facet contacting the navicular facet, would also facilitate the inversion and eversion of the pes [20] during swimming. The pFDA demonstrates that although the influence of evolutionary history on astragalar morphology is small, the consideration of phylogenetic relationships substantially improves the classification of taxa, particularly in ricochetal, fossorial and semi-aquatic taxa. This may be a product of the evolution of these specialized ecologies in a limited number of taxa across the tree. All analyses show strong support for a semi-aquatic ecology for *Microtheriomys articulaquaticus* sp. nov. and a terrestrial ecology for *Eutypomys thomsoni*.

The sedimentology and fossil record of the six localities where *Microtheriomys articulaquaticus* sp. nov. has been recovered support its amphibious ecology. At all localities where *M. articulaquaticus* sp. nov. has been found, abundant freshwater snails and frog remains are also present [64–66]. At UWBM C1708, the lowest locality stratigraphically where the animal is recovered, abundant fish have also been recovered; the environment was ponded, at least periodically [64]. The sedimentology and palaeontology of UWBM C1707 suggest the presence of a high-energy river channel [64]. The sedimentology and taphonomy of MV6554, C1708 and C1721 support an aquatic environment with some current [30,67]. In general, the depositional environments of the lower Cabbage Patch, where most specimens of *M. articulaquaticus* sp. nov. have been recovered, likely represent water bodies in a forested mesic environment [64]. The single locality of the middle Cabbage Patch beds where *Microtheriomys* has been recovered (UWBM C1706) includes a very thick shell-rich diatomite deposit as well as reworked ash layers indicative of a large pond or lake.

Radioisotopic analyses of the Cabbage Patch beds demonstrate that the sediments encasing the oldest specimens of *Microtheriomys articulaquaticus* sp. nov. date to 29.92 ± 0.07 Ma [30]. The existence of a semi-aquatic rodent in North America at that time pushes back the first occurrence of this ecology on the continent by over 11 Myr to the start of the Arikareean North American Land Mammal Age. This time period is associated with immigration events from Eurasia, particularly in the western United States, where *Microtheriomys* is recorded [30].

*Microtheriomys* represents the oldest confirmed semi-aquatic castorid in the world. Prior to this analysis, the oldest semi-aquatic beaver was *Steneofiber eseri* from the early Miocene (approx. 23 Ma) of France [9]. *Steneofiber* was classified as semi-aquatic based on a specialized ungual phalanx. The usefulness of the astragalus in identifying both fossorial and semi-aquatic behaviours will facilitate the identification of the locomotory ecologies of other castorids across the world and therefore the analysis of locomotory evolution in the family.

The evolution of semi-aquatic locomotion in Castoridae has been associated with the evolution of woodcutting behaviour and dated to *circa* 24 Ma in the common ancestor of the extant beaver, *Castor*, and the extinct *Dipoides* [10,68,69]. Several lines of evidence including trace fossils, isotope data and ancient DNA have been used to support a scenario whereby a semi-aquatic ecology associated with woodcutting and a woody-plant diet evolved from a terrestrial/burrowing ancestor in the clade comprising Castorinae and Castoroidinae, dam building evolved in *Castor*, and the woody-plant diet was lost in *Castoroides* [68,70,71]. The presence of a semi-aquatic beaver during the Oligocene enables a revision of this paradigm. Indeed, my ancestral character state reconstruction of locomotion supports a better understanding of castorid evolution in which semi-aquatic locomotion evolved during the Oligocene several million years earlier in the common ancestor of Anchitheriomyinae, Castorinae and Castoroidinae. It is unknown whether *Microtheriomys* could engage in woodcutting. Future analyses of incisor microstructure will be necessary to rigorously test this aspect of ecology; the semi-flattened lower incisor of *Microtheriomys*, intermediate in shape *between Dipoides* and *Castor*, does not help resolve its woodcutting ability. However, the semi-aquatic locomotion of *Microtheriomys* could have evolved independently from woodcutting. Indeed, data from *Castoroides* demonstrate that the two ecologies can be decoupled [71] and the study of *Dipoides* shows that it was not as effective a woodcutter as the extant *Castor* [70]. Thus, woodcutting may have been perfected over time after the evolution of amphibious behaviour. The locomotory ecology of additional anchitheriomyines will have to be analysed to confirm the hypothesis laid out herein. However, already, *Anchitheriomys tungurensis* from the middle Miocene of China has been suggested to be semi-aquatic [11,72]. If all anchitheriomyines were semi-aquatic and the relatively poorly known fossil beaver *Agnotocastor* is indeed a junior synonym of *Propalaeocastor*, the oldest anchitheriomyine in the world would be *P. galushai* from the early Oligocene of North America [73]. This would suggest the evolution of amphibious beavers first in North America, early in the Oligocene, rather than in Eurasia during the Miocene as currently thought. Amphibious beavers could then have emigrated to Eurasia at the same time that numerous mammal taxa immigrated to North America during the Arikareean through Beringia [30]. Future phylogenetic and biogeographic analyses will be necessary to test this hypothesis.

My work supports the hypothesis that the evolution of the semi-aquatic ecology of beavers is an exaptation from the fossorial ecology of the common ancestor of Castoridae. Patterns of exaptation between burrowing and swimming have been identified or hypothesized in other mammalian clades [74,75]. The relative ease by which one mode of locomotion can evolve from the other is likely facilitated by their many similarities in associated morphology and biomechanics [76], including in Rodentia [5]. This evolutionary pattern may explain the second likely classification of fossoriality for *Microtheriomys*; the animal bears the morphology inherited from its ancestors while displaying evidence of divergence away from burrowing towards swimming. Alternatively, or concurrently, the second likely classification of fossoriality for *Microtheriomys* may reflect its ability to burrow retained from its ancestors; the extant *Castor* is indeed capable of extensive digging [12]. Additional fossils of the forelimb and/or skull of *Microtheriomys* will be necessary to further test this hypothesis. The evolutionary success of semi-aquatic beavers when their fossorial relatives were waning during the early to mid-Miocene [52] supports the hypothesis that the survival and success of the family were achieved through the exaptation of a morphology adapted to moving through dirt for locomotion through water as habitats were changing with increased heterogeneity [7]. Further analyses of morphological similarities and differences among semi-aquatic rodents across families may reveal important evidence for canalization and help constrain the processes of exaptation in Castoridae.

Extant semi-aquatic beavers are the second largest rodent in the world today; the largest extant rodent, the capybara, is amphibious; the largest beavers to have existed were semi-aquatic; the largest known rodents ever found had amphibious ecologies [8,31,77,78]. Yet, there is evidence for many small semi-aquatic beavers in the fossil record, including now *Microtheriomys*, the fifth smallest amphibious beaver to date. Interestingly, the previously oldest semi-aquatic North American beaver, *Monosaulax*, is also among the smallest five amphibious beavers; *Eucastor tedi*, another Miocene-aged amphibious beaver, is also small [52]. Together, these data suggest that the semi-aquatic ecology of Castoridae first evolved in small species with the gigantism of younger species evolving later. This is consistent with the pattern described above of an exaptation from fossoriality; burrowing beavers (Palaeocastorinae) are smaller than semi-aquatic ones. The ancestral character reconstruction and the model fitting analysis corroborate this inference. The directional drift followed by body size in castoroids supports the hypothesis that beavers followed Cope's rule, alike canids [79]. It may even be that the evolution of large body sizes drove beavers, like muskrats, another semi-aquatic rodent group, to their current low diversity of two extant species each (*Castor fiber* and *C. canadensis*, *Neofiber alleni* and *Ondatra zibethicus*, respectively) because of a possible inverse relationship between body mass and species diversity [29]. Future comparative phylogenetic analyses sampling a greater portion of the Castoridae will be necessary to rigorously test the hypothesis that the semi-aquatic locomotion of beavers acquired during the Oligocene played a role in the evolution of some of the largest rodent species in the world during the Pliocene and Pleistocene.

## Data Availability

The data used in the quantitative analyses of locomotion are included in the electronic supplementary material. Toothrow length data are provided in the text and publications cited. The nexus file for the supertree and the code used for the analyses are provided in the electronic supplementary material [80].
